# A clinically used anti‐human papilloma virus agent (3‐hydroxyphthalic anhydride‐modified bovine β‐lactoglobulin) has a potential for topical application to prevent sexual transmission of monkeypox virus

**DOI:** 10.1002/mco2.677

**Published:** 2024-08-04

**Authors:** Yi'ou Sha, Baoying Huang, Chen Hua, Yun Zhu, Wanbo Tai, Jiewei Sun, Yixin Li, Anqi Xia, Qiao Wang, Lu Lu, Wenjie Tan, Shibo Jiang

**Affiliations:** ^1^ Key Laboratory of Medical Molecular Virology (MOE/NHC/CAMS) Shanghai Institute of Infectious Disease and Biosecurity School of Basic Medical Sciences Fudan University Shanghai China; ^2^ National Key Laboratory of Intelligent Tracking and Forecasting for Infectious Diseases (NITFID) NHC Key Laboratory of Biosafety National Institute for Viral Disease Control and Prevention Chinese Center for Disease Control and Prevention Beijing China; ^3^ Shanxi Key Laboratory of Functional Proteins, Fudan‐Jinbo Functional Protein Joint Research Center Shanxi Jinbo Bio‐Pharmaceutical Co. Ltd. Taiyuan Shanxi China; ^4^ National Laboratory of Biomacromolecules Institute of Biophysics Chinese Academy of Sciences Beijing China; ^5^ Institute of Infectious Diseases Shenzhen Bay Laboratory Shenzhen China

**Keywords:** antiviral agent, monkeypox virus, sexual transmission, vaccinia virus Tiantan strain

## Abstract

A global outbreak of monkeypox (mpox) caused by the mpox virus (MPXV) has posed a serious threat to public health worldwide, thus calling for the urgent development of antivirals and vaccines to curb its further spread. In this study, we screened 41 anhydride‐modified proteins and found that 3‐hydroxyphthalic anhydride‐modified β‐lactoglobulin (3HP‐β‐LG), a clinically used anti‐HPV agent, was highly effective in inhibiting infection of vaccinia virus Tiantan strain (VACV‐VTT) and MPXV. Mechanistic studies demonstrated that 3HP‐β‐LG bound to the virus, not the host cell, by targeting the early stage of virus entry, possibly through the interaction between the amino acids with negatively charges in 3HP‐β‐LG and the key amino acids with positive charges in the target region of A29L, a key surface protein of MPXV. A synergistic effect was observed when 3HP‐β‐LG was combined with tecovirimat, a small‐molecule antiviral drug approved by the United States Food and Drug Administration and the European Medicine Agency for the treatment of smallpox and mpox. Because of its clinically proven safety and stability, 3HP‐β‐LG shows promise for further development as a prophylactic agent to prevent the sexual transmission of MPXV.

## Graphical abstract

This study has demonstrated that 3‐hydroxyphthalic anhydride‐modified β‐lactoglobulin (3HP‐β‐LG), a clinically used anti‐HPV agent, is effective in inhibiting infection of MPXV, possibly by targeting surface protein A29L of MPXV. Because of its clinically proven safety and stability, 3HP‐β‐LG shows promise for further development as a prophylactic agent to prevent the sexual transmission of MPXV

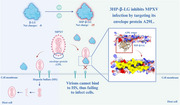

## 1 INTRODUCION

Monkeypox (mpox), is a viral illness that spreads from animals to humans and is caused by the monkeypox virus (MPXV) infection. A global outbreak of MPXV started in May 2022 in Europe. From there, it rapidly spread across the world. The outbreak and spread of MPXV call for the urgent development of antivirals and vaccines.^1^ International Concern by the World Health Organization has declared MPXV a Public Health Emergency on July 23, 2022. As the disease trajectory evolved and understanding deepened, a strategic shift toward a sustained and long‐term approach was formalized on May 11, 2023 (https://www.who.int/news/item/11‐05‐2023‐fifth‐meeting‐of‐the‐international‐health‐regulations‐(2005)‐(ihr)‐emergency‐committee‐on‐the‐multi‐country‐outbreak‐of‐monkeypox‐(mpox)). Sexual transmission is the main route of MPXV dissemination. The ongoing mpox epidemic seems to primarily be spreading within distinct sexual networks involving gays, bisexual individuals, or men who engage in sexual activity with other men.^2^ Thus, it is essential to develop a locally applicable antiviral for prevention of sexual transmission of MPXV.

The orthopoxvirus (OPXV) genus of the family Poxviridae includes MPXV, variola virus, vaccinia virus (VACV), cowpox virus, and others.^3^ Infection of these OPXVs can induce cross‐immunological protection in the infected hosts, indicating that infection with one of the OPXVs may confer substantial immunity against infection by other members of the same genus.^4^ VACV Tiantan strain (VACV‐VTT) has served as the antigenic basis for smallpox vaccines, playing a pivotal role in eradicating smallpox worldwide.^5^ Currently, smallpox vaccination has been employed for individuals who have exposed to or have been in close contact with MPXV in certain high‐risk areas, showing efficacy in preventing MPXV infection.^6^ Given the genetic and antigenic similarities shared among these OPXVs,^4^ antivirals capable of inhibiting VACV‐VTT infection may also be effective against MPXV infection.

Cidofovir, brincidofovir, and tecovirimat have been recognized as effective antiviral drugs against OPXV infection.^7^ Cidofovir and brincidofovir both function by suppressing DNA polymerase activity,^8^ yet brincidofovir has shown gastrointestinal and hepatic toxicities in clinical trials, rendering it less safe compared with tecovirimat.^9^ Tecovirimat interacts with the orthopoxviral protein p37, effectively obstructing intercellular viral dissemination.^10^ However, few rigorously evidenced prophylactic and therapeutic options are now available for MPXV infection,^11^ even while drug‐resistant OPXVs have emerged as a consequence of viral mutation, possibly making existing medications ineffective against such strains in the long run. Thus, it is essential to identify antivirals with different mechanisms of action against infection by MPXV and resistance to viral mutations of OPXVs that may emerge in the near future.

We have previously identified 3‐hydroxyphthalic anhydride‐modified β‐lactoglobulin (3HP‐β‐LG) from 62 chemically modified milk proteins as an anti‐ human immunodeficiency virus type 1 (HIV‐1) agent for the prevention of sexually transmitted HIV‐1.^12^, ^13^ Later, we found that 3HP‐β‐LG was also effective in inhibiting infection of human papilloma virus (HPV),^14^, ^15^ severe acute respiratory syndrome coronavirus 2 (SARS‐CoV‐2),^16^ and influenza virus.^17^ Particularly, a 3HP‐β‐LG‐containing biological dressing (JB01‐BD) was approved in 2012 to block cervical infection of HPV,^18^ which reminds us that this locally applicable formulation could also be used to prevent the sexual transmission of MPXV if 3HP‐β‐LG is also demonstrated to efficaciously counteract MPXV infections.

In this study, we found that 3HP‐β‐LG effectively inhibited VACV‐VTT and MPXV infection by blocking their entry into the host cells, possibly through its interaction with the surface protein A29L of MPXV. Furthermore, we observed a synergistic effect when 3HP‐β‐LG was combined with tecovirimat. Additionally, we found that JB01‐BD, the 3HP‐β‐LG‐containing biological dressing,^18^ exhibited pronounced inhibitory activity against VACV‐VTT infection, implying that it harbors the potential to be used to prevent the sexual transmission of MPXV.

## 2 RESULTS

### 2.1 Identification of 3HP‐β‐LG as an inhibitor of VACV‐VTT and MPXV infection

**FIGURE 1:**
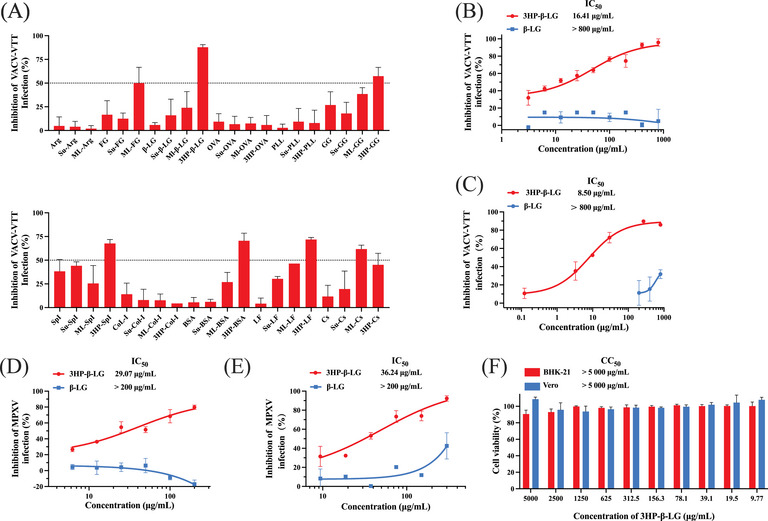
Identification of 3HP‐β‐LG as an inhibitor of VACV‐VTT and MPXV infection. (A) Inhibitory activity of anhydride‐modified proteins at a concentration of 200 µg/mL against VACV‐VTT infection was evaluated using a plaque‐reduction assay. Each sample was tested in triplicate, and the experiment was repeated at least twice. The results from a representative experiment were presented in means ± standard deviation (SD). Inhibitory activity of 3HP‐β‐LG and β‐LG against VACV‐VTT infection was evaluated with a plaque‐reduction assay (B) and qPCR assay (C). Inhibitory activity of 3HP‐β‐LG and β‐LG against MPXV infection was evaluated using a plaque‐reduction assay (D) and qPCR assay (E). (F) Cytotoxic effect of 3HP‐β‐LG at concentrations varying from 9.77 to 5000 µg/mL on BHK‐21 and Vero cells was assessed using CCK‐8 kit.

In order to identify an anti‐MPXV agent, a plaque‐reduction assay was used to detect the inhibitory activity of 41 anhydride‐modified proteins (Table 1) on infection of VACV‐VTT (GenBank: AF095689.1), which belongs to the same poxviridae family as MPXV. We found that six of these anhydride‐modified proteins, including 3HP‐β‐LG, 3HP‐modified bovine serum albumin (3HP‐BSA), gamma globulin (3HP‐GG), lactoferrin (3HP‐LF), and soybean protein isolate (3HP‐SpI), as well as maleic anhydride (ML)‐modified casein (ML‐Cs), all showed over 50% inhibition at the concentration of 200 µg/mL (Figure 1A). Among them, 3HP‐β‐LG was the most effective in inhibiting VACV‐VTT infection in BHK‐21 cells. Considering that 3HP‐β‐LG has been safely used in the clinic, we selected it for further study. We then retested the inhibitory effects of 3HP‐β‐LG at graded concentration, starting from 800 µg/mL, and found that it inhibited VACV‐VTT infection in a dose‐dependent manner with the concentration required to achieve half‐maximal inhibition (IC_50_) at 16.41 ± 1.68 µg/mL, while β‐LG exhibited no detectable inhibitory activity at concentrations up to 800 µg/mL (Figure 1B). We then performed a quantitative real‐time polymerase chain reaction (qPCR) assay as previously described^19^ to assess the infectivity of VACV‐VTT. This assay is used to detect the genes encoding the VACV surface proteins E9L. Since the protein possesses conserved amino acid sequences among OPXVs, it is useful as benchmark for detecting the load of OPXV. Results showed that 3HP‐β‐LG inhibited VACV‐VTT infection in a dose‐dependent manner with IC_50_ of 8.50 ± 2.21 µg/mL (Figure 1C).

**TABLE 1:** Anhydride‐modified proteins used for screens of inhibitory activity.

Abbreviation	Full name
Arg	Arginine
Su‐Arg	Succinic anhydride‐modified arginine
ML‐Arg	Maleic anhydride‐modified arginine
FG	Fibrinogen
Su‐FG	Succinic anhydride‐modified fibrinogen
ML‐FG	Maleic anhydride‐modified fibrinogen
β‐LG	β‐Lactoglobulin
Su‐β‐LG	Succinic anhydride‐modified β‐lactoglobulin
ML‐β‐LG	Maleic anhydride‐modified β‐lactoglobulin
3HP‐β‐LG	3‐Hydroxyphthalic anhydride‐modified β‐lactoglobulin
OVA	Ovalbumin
Su‐OVA	Succinic anhydride‐modified ovalbumin
ML‐OVA	Maleic anhydride‐modified ovalbumin
3HP‐OVA	3‐Hydroxyphthalic anhydride‐modified ovalbumin
PLL	Poly‐l‐lysine
Su‐PLL	Succinic anhydride‐modified poly‐l‐lysine
3HP‐PLL	3‐Hydroxyphthalic anhydride‐modified poly‐l‐lysine
GG	Gamma globulin
Su‐GG	Succinic anhydride‐modified gamma globulin
ML‐GG	Maleic anhydride‐modified gamma globulin
3HP‐GG	3‐Hydroxyphthalic anhydride‐modified gamma globulin
SpI	Soybean protein isolate
Su‐SpI	Succinic anhydride‐modified soybean protein isolate
ML‐SpI	Maleic anhydride‐modified soybean protein isolate
3HP‐SpI	3‐Hydroxyphthalic anhydride‐modified soybean protein isolate
Col‐I	Collagen‐I
Su‐Col‐I	Succinic anhydride‐modified collagen‐I
ML‐Col‐I	Maleic anhydride‐modified collagen‐I
3HP‐Col‐I	3‐Hydroxyphthalic anhydride‐modified collagen‐I
BSA	Bovine serum albumin
Su‐BSA	Succinic anhydride‐modified bovine serum albumin
ML‐BSA	Maleic anhydride‐modified bovine serum albumin
3HP‐BSA	3‐Hydroxyphthalic anhydride‐modified bovine serum albumin
LF	Lactoferrin
Su‐LF	Succinic anhydride‐modified lactoferrin
ML‐LF	Maleic anhydride‐modified lactoferrin
3HP‐LF	3‐Hydroxyphthalic anhydride‐modified lactoferrin
Cs	Casein
Su‐Cs	Succinic anhydride‐modified casein
ML‐Cs	Maleic anhydride‐modified casein
3HP‐Cs	3‐Hydroxyphthalic anhydride‐modified casein

Subsequently, plaque‐reduction assay and qPCR were used to test the inhibitory activity of 3HP‐β‐LG and β‐LG against MPXV infection, and we found that 3HP‐β‐LG inhibited MPXV infection in a dose‐dependent manner with IC_50_ of 29.07 ± 4.87 µg/mL, while β‐LG exhibited no inhibitory effect at concentrations up to 200 µg/mL (Figure 1D). We then performed qPCR by targeting F3L, which was used as a target to detect MPXV infection.^20^ 3HP‐β‐LG could effectively inhibit MPXV infection with IC_50_ of 36.24 ± 4.31 µg/mL, whereas β‐LG showed no significant inhibition at concentrations up to 300 µg/mL (Figure 1D).

According to our previous report, 3HP‐β‐LG exhibits no cytotoxicity to common human cell lines.^17^ Here, as confirmation of this, we evaluated the cytotoxicity of 3HP‐β‐LG to BHK‐21 and Vero cells using the Cell Counting Kit‐8 (CCK‐8) assay and found that 3HP‐β‐LG also exhibited no cytotoxicity at concentrations as high as 5000 µg/mL (Figure 1F).

### 2.2 Potential mechanism of 3HP‐β‐LG underlying inhibition of VACV‐VTT and MPXV infection

**FIGURE 2:**
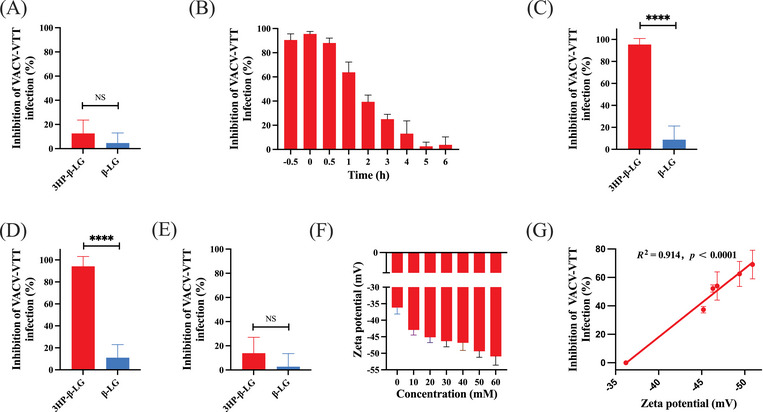
Potential mechanism of action of 3HP‐β‐LG against VACV‐VTT infection. (A) Inhibitory activity of 3HP‐β‐LG and β‐LG at a concentration of 800 µg/mL against VACV‐VTT infection was evaluated by cell‐washout assay. Each sample was tested in triplicate, and the experiment was repeated at least twice. The results from a representative experiment were presented in means ± SD. Student's *t*‐test and Analysis of Variance (ANOVA) were used to compare the difference by GraphPad Prism 10.0. *p < 0.05, **p < 0.01, ***p < 0.001, and ****p < 0.0001, NS, no significant difference. (B) Time‐of‐addition assay for comparing the inhibitory potency of 3HP‐β‐LG against VACV‐VTT infection when 3HP‐β‐LG was added at −0.5 h (half an hour before), immediately (0 h), and 0.5–6 h after addition of VACV‐VTT. Determining the mechanism by which 3HP‐β‐LG inhibits VACV‐VTT infection by binding assay (C), postattachment assay (D) and postentry assay (E). (F) Zeta potential of 3HP‐β‐LG with varying degrees of modification. (G) Correlation between the inhibitory activity of 3HP‐β‐LG and its zeta potentials against VACV‐VTT infection.

Our previous research has shown that 3HP‐β‐LG impedes viral entry by halting the ingress of virions into host cells.^15^, ^16^, ^17^ Based on this, we employed similar approaches to elucidate how 3HP‐β‐LG inhibits VACV‐VTT infection specifically. First, we carried out a cellular washout experiment to determine whether the inhibition of VACV‐VTT by 3HP‐β‐LG acts at the cellular level or targets the virions directly. This pretreating BHK‐21 cells with 3HP‐β‐LG at 37°C for an hour, followed by washing cells with culture medium before adding virus. No significant inhibitory effect was detected after unbound 3HP‐β‐LG was washed away from cells (Figure 2A), suggesting that 3HP‐β‐LG does not interact with cells, but does possibly interact with virions.

Poxvirus entry into cells typically occurs within 1 h, which is a notably rapid process compared with other viruses.^21^ To determine when 3HP‐β‐LG blocks poxvirus entry into cells, a time‐of‐addition assay was carried out. The result showed that 3HP‐β‐LG could effectively block VACV‐VTT infection when added to cells at 0.5 h before or 0, 0.5, and 1 h after the addition of virus, while its inhibitory activity decreased significantly at 2–6 h after virus addition (Figure 2B), indicating that 3HP‐β‐LG mainly works at the early stage of VACV‐VTT infection.

To determine the exact stage of viral entry blocked by 3HP‐β‐LG, we slowed down the process of viral infection by adjusting temperature. At 4°C, virions could bind to the cell surface, but could not complete membrane fusion, thus preventing the virus from fully entering the cytoplasm. 3HP‐β‐LG alongside β‐LG as a control was combined with VACV‐VTT at 4°C for 1 h. Following this, cells were washed to remove unbound virions and inhibitor and then cultured at 37°C until plaques became visible. Our finding revealed that 3HP‐β‐LG exhibited inhibitory activity against VACV‐VTT infection, but β‐LG failed to do so, indicating that 3HP‐β‐LG interacts with virus and blocks its binding to cells, which is the earliest phase of the virus life‐cycle (Figure 2C).

In another experiment, virus was incubated with cells at 4°C for 1 h, allowing virus to bind cell surface receptor. Then, cells were washed to remove the unbound virus before addition of 3HP‐β‐LG or β‐LG, followed by incubation at 37°C until plaques appeared. 3HP‐β‐LG added after initial virus exposure still exhibited inhibitory activity, while β‐LG did not, suggesting that 3HP‐β‐LG can also bind the virus that had already attached to cells via protein receptor, thus still blocking viral entry into the cell (Figure 2D).

Finally, we incubated both cells and VACV‐VTT at 37°C for 1 h, allowing viral entry into cells. Then, the cells were washed and incubated with 3HP‐β‐LG, or β‐LG, at 37°C until plaques were visible. This time, neither 3HP‐β‐LG nor β‐LG showed significant inhibition (Figure 2E), indicating that once virus had entered the cell, 3HP‐β‐LG lost its inhibitory activity against virus replication in cells and again confirming that 3HP‐β‐LG acts at the early stage of viral entry, rather than the postentry stage of virus.

Our earlier research has demonstrated a positive association between the concentration of 3HP employed for protein alteration and the antiviral activity of the modified protein.^15^, ^16^ Specifically, the process of 3HP modification leads to a decrease in the count of positively charged amino acids in β‐LG, yielding an increase of net negative charges.^17^ Consequently, 3HP‐β‐LG possesses the capability to bind with positively charged amino acids located within the viral surface protein target area, effectively obstructing viral entry and subsequent infection.^17^ Here, we also used different concentrations of 3HP to modify β‐LG, followed by detecting surface charges by zeta potential and inhibitory activity. By increasing the degree of anhydride, the negative charges on the surface of 3HP‐β‐LG and its inhibitory activity were increased (Figure 2F,G). These results are consistent with those from the studies of 3HP‐β‐LG against HPV and SARS‐CoV‐2 infection.^15^, ^16^

### 2.3 Binding of 3HP‐β‐LG to the surface protein A29L of MPXV

**FIGURE 3:**
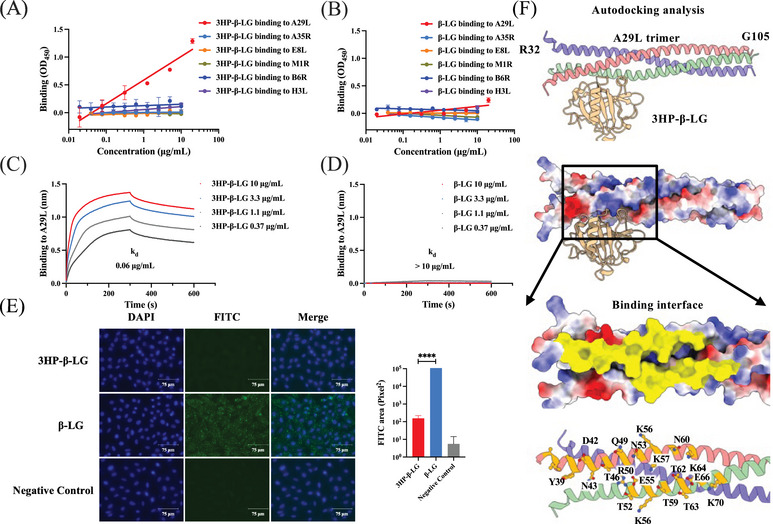
Potential interaction of 3HP‐β‐LG with MPXV surface proteins. (A) Binding of 3HP‐β‐LG to MPXV surface proteins A29L, A35R, B6R, E8L, H3L, and M1R detected with ELISA. Briefly, 15 µg/mL 3HP‐β‐LG or β‐LG were coated on wells of a 96‐well plate, and MPXV surface protein at graded concentration was added for the detection of binding affinity. (B) Binding of β‐LG to MPXV surface proteins A29L, A35R, B6R, E8L, H3L, and M1R detected with ELISA. (C) Binding affinity of 3HP‐β‐LG to MPXV protein A29L detected by BLI. (D) Binding affinity of β‐LG to MPXV protein A29L detected by BLI. (E) Inhibition of 3HP‐β‐LG or β‐LG on binding of A29L to Vero cells. A29L was preincubated with 3HP‐β‐LG or β‐LG before being added to Vero cells, and A29L binding to cells was detected by immunofluorescence assay using anti‐A29L hAb (green). Cell nuclei were stained by 4,6‐diamidino‐2‐phenylindole (blue). (F) Molecular docking simulation using AutoDock suggests that 3HP‐β‐LG interacts with the Y39‐K70 region in MPXV A29L protein.

In order to study the mechanism by which 3HP‐β‐LG inhibits MPXV infection, we examined its binding efficacy with MPXV surface proteins, including A29L, E8L, H3L, and M1R on the intracellular mature viral (IMV) particle, as well as A35R and B6R on the extracellular envelope viral form.^22^, ^23^ Among them, A29L, H3L, and E8L exhibited strong affinity for glycosaminoglycans (GAGs) that mediate the entry of MPXV into target cells. We found that 3HP‐β‐LG bound to A29L, but no other viral surface proteins (Figure 3A), while β‐LG bound none of these MPXV surface proteins (Figure 3B). Using biolayer interferometry (BLI), we demonstrated that the binding affinity of 3HP‐β‐LG to A29L was 0.06 µg/mL, while β‐LG showed no detectable binding affinity at concentrations as high as 10 µg/mL (Figure 3C,D). A29L shows high binding affinity to heparan sulfate proteoglycan (HSPG), which is a type of GAG.^24^ We then used an immunofluorescence assay to determine the binding of A29L pretreated with 3HP‐β‐LG or β‐LG to Vero cells that express HS.^25^ As shown in Figure 3E, the number of A29L‐bound Vero cells was remarkably decreased in the presence of 3HP‐β‐LG, suggesting that 3HP‐β‐LG can block the attachment of A29L to Vero cells, thus inhibiting MPXV infection in these susceptible cells. Results from the molecular simulation analysis with AutoDock reveal that 3HP‐β‐LG interacts with the Y39‐K70 region of A29L (Figure 3F).

### 2.4 Synergistic anti‐VACV‐VTT effect of 3HP‐β‐LG in combination with tecovirimat and the effectiveness of 3HP‐β‐LG‐containing lubricant against VACV‐VTT infection

As mentioned above, tecovirimat, a small‐molecule antiviral medication, has attained approval from both the United States Food and Drug Administration and the European Medicine Agency for the therapy of smallpox and mpox viral infections. Tecovirimat specifically targets the VP37 envelope coating protein, an indispensable component for OPXVs that facilitates the envelopment of IMVs with membranes derived from the Golgi apparatus to generate intracellular enveloped viruses, thus obstructing the ultimate stage of viral maturation and release and hindering the virus’ propagation within the host organism.^26^ Considering that 3HP‐β‐LG primarily acts on the viral entry stage with a different mechanism of action from that of tecovirimat, we tested the potential synergistic effect when 3HP‐β‐LG was combined with tecovirimat. As illustrated in Figure 3, 4 HP‐β‐LG and tecovirimat in combination was about 10‐ and 30‐fold more potent against VACV‐VTT infection than 3HP‐β‐LG and tecovirimat tested alone, respectively. The combination index (CI) was 0.134 (Table 2), suggesting that the 3HP‐β‐LG/tecovirimat combination exhibits a strong synergistic effect in inhibiting VACV‐VTT infection.

**FIGURE 4:**
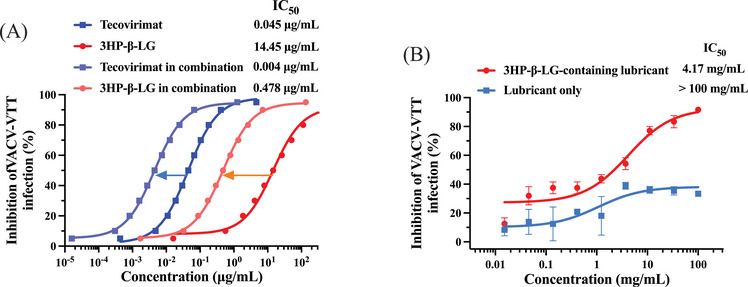
Potential application of 3HP‐β‐LG in combination with tecovirimat or in lubricant against VACV‐VTT infection. (A) Synergistic anti‐VACV‐VTT effect achieved by combining 3HP‐β‐LG with tecovirimat. (B) Inhibitory effect of 3HP‐β‐LG‐containing lubricant against VACV‐VTT infection. Each sample was tested in triplicate, and the experiment was repeated at least twice. The results from a representative experiment were presented in means ± standard deviation (SD).

**TABLE 2:** Combination index (CI) and dose reduction values of inhibiting 50% PsV infection by 3HP‐β‐LG in combination with tecovirimat against VACA‐VTT.

Inhibitor A	IC50 (µg/mL)		Dose	Inhibitor B	IC50 (µg/mL)		Dose	CI
	Alone	In combination	Reduction (fold)		Alone	In combination	Reduction (fold)	
Tecovirimat	0.045	0.004	9.92	3HP‐β‐LG	14.451	0.478	30.21	0.134

*Note*: A combination index (CI) exceeding 1 signifies antagonism, equality to 1 suggests an additive effect, while a value less than 1 denotes synergism. Moreover, the strength of synergistic effect is inferred by the CI value, where lower values denote a stronger synergism.

Next, we evaluated the anti‐VACV‐VTT activity of a vaginally applied 3HP‐β‐LG‐containing lubricant, originally designed as an anti‐HPV product developed by Jinbo Bio‐Pharmaceutical Co., Ltd., Taiyuan, China (http://en.sxjbswyy.com). As illustrated in Figure 4B, 3HP‐β‐LG in this topical formulation inhibited VACV‐VTT infection in a dose‐dependent manner with IC_50_ of about 2 mg/mL, suggesting that this 3HP‐β‐LG‐containing lubricant is a promising candidate for further development as a locally applicable antiviral product for the prevention of sexually transmitted MPXV.

## 3 DISSCUSSION

Our previous studies have shown that 3HP‐β‐LG potently inhibits HIV‐1 infection by binding to CD4 receptor on host cell, thus blocking the attachment of HIV‐1 virions to the CD4+ cells.^13^ Later, we found that 3HP‐β‐LG was also capable of inhibiting infection of HPV,^14^, ^15^ SARS‐CoV‐2,^16^ and influenza virus^17^ by targeting the virions, rather than the cells, through the interaction between regions in 3HP‐β‐LG that are rich in negatively charged amino acids and corresponding regions containing key amino acids with positive charge in some viral surface proteins. Similarly, the results from this study suggest that 3HP‐β‐LG inhibits infection of VACV‐VTT and MPXV by also targeting virions, not cells, possibly through binding of 3HP‐β‐LG to A29L, a key surface protein of MPXV. A previous report has shown that A29L could bind HSPG, a type of GAG, with high affinity.^24^ Therefore, 3HP‐β‐LG may suppress the binding of A29L to HSPG through its interaction with A29L, resulting in the failure of MPXV to gain entry into host cells. Molecular docking simulation using AutoDock suggests that 3HP‐β‐LG interacts with the Y39‐K70 region in MPXV A29L protein (Figure 3F). The mechanistic study indicates that 3HP‐β‐LG acts at the early stage of viral entry, rather than the postentry stage (Figure 2B–E). Consistent with our previous reports,^15^, ^16^, ^17^ the quantity of negative charges present in 3HP‐β‐LG is correlated with the concentration of 3HP utilized during the β‐LG modification process (Figure 2F), which in turn aligns with the terminal inhibitory capacity of 3HP‐β‐LG (Figure 2G). This relationship implies that the antiviral effectiveness of 3HP‐β‐LG is fundamentally linked to electrostatic interactions with proteins on viral surface. Moreover, conformation of the target region in viral protein must match conformation of the binding region in 3HP‐β‐LG, allowing their close contact and interaction.

The mpox outbreak report from the European Centre for Disease Prevention and World Health Organization (WHO) states that MPXV has mainly spread through male‐to‐male sexual transmission (https://worldhealthorg.shinyapps.io/mpx_global). Therefore, the development of an antiviral agent to block male‐to‐male sexual transmission of MPXV is urgently needed. Our previous studies have shown that 3HP‐β‐LG is highly effective against HIV and HPV infection and that it can be used to prevent the sexual transmission of HIV and HPV.^13^, ^14^, ^15^, ^27^ In collaboration with a group of researchers from six hospitals in China, we have successfully developed a 3HP‐β‐LG‐containing anti‐HPV biological dressing (JB01‐BD) to suppress cervical infection of HPV,^18^ and a 3HP‐β‐LG‐containing lubricant to prevent the sexual transmission of HPV. In this study, we found that a 3HP‐β‐LG‐containing lubricant was also effective in inhibiting VACV‐VTT infection, suggesting that this 3HP‐β‐LG‐containing lubricant may be used to prevent the sexual transmission of MPXV.

Tecovirimat (ST‐246) has been used clinically for the treatment of mpox,^2^ and a clinical study has shown that the currently circulating MPXV remains sensitive to tecovirimat.^7^ Different from 3HP‐β‐LG that blocks MPXV entry into the host cells by targeting A29L, tecovirimat inhibits MPXV infection by targeting the orthopoxviral protein p37 to interfere with intercellular viral dissemination.^10^ With good oral bioavailability, tecovirimat could be conveniently used for treatment of MPXV infection. However, its use in the prevention of sexually transmitted MPXV is impractical because only very little portion of the drug that is systemically absorbed will be distributed into the rectal or vaginal mucosa where the virus is sexually transmitted, while most of the absorbed compound is circulating in other organs or tissues, which may not be useful for local prevention of sexual transmission of MPXV. However, this study has shown that the combinational use of 3HP‐β‐LG and tecovirimat exhibited a strong synergistic effect (CI = 0.134) in inhibiting VACV‐VTT infection. Therefore, 3HP‐β‐LG used alone or in combination with tecovirimat in a lubricant formulated for rectal or vaginal application is plausible to prevent male‐to‐male or male‐to‐female sexual transmission of MPXV, respectively. It is speculated that the local use of a 3HP‐β‐LG/tecovirimat topical formulation could also be effective in preventing infection of other OPXVs through sexual transmission.

## 4 MATERIALS AND METHODS

### 4.1 Cell lines, viruses, and proteins

The baby hamster kidney cell line BHK‐21 (catalogued as GNHa10) and the African green monkey kidney epithelial cell line Vero (designated as GNO10) were both obtained from the National Collection of Authenticated Cell Cultures. The Authentic MPXV strain MPXV‐B.1‐China‐C‐Tan‐CQ01 was isolated from genuine clinical specimens^28^ and maintained at the NHC Key Laboratory of Biosafety, Chinese Center for Disease Control and Prevention. The VACV‐VTT strain was maintained at the Institute of Infectious Diseases, Shenzhen Bay Laboratory.

### 4.2 Primers and probes

Primers and probes targeting the loci for VACV‐VTT‐E9L and MPXV‐F3L^28^ were synthesized by Thermo Fisher.

Sequences used were as follows:

E9L forward 5′‐GTCCAACGAGTAACATCCGTCTGG‐3′,

E9L reverse 5′‐GCTCCGTCGCAGATATGTGGTTG‐3′.

F3L forward 5′´‐CATCTATTATAGCATCAGCATCAGA‐3′,

F3L reverse 5′‐GATACTCCTCCTCGTTGGTCTAC −3′,

F3L probe 5′‐FAM/TGTAGGCCGTGTATCAGCATCCATT/BHQ1‐3′.

### 4.3 Preparation and characterization of 3HP‐β‐LG

The chemically modified proteins (Table 1) were prepared as previously described.^12^, ^15^, ^16^ For preparation of 3HP‐β‐LG, the bovine whey protein β‐LG (Sigma–Aldrich; product #L8005) was subjected to modification using 3‐hydroxyphthalic anhydride (3HP) (Sigma–Aldrich; product #37418‐88‐5). Briefly, β‐LG was solubilized to 10 mg/mL in a 0.1 M dibasic sodium phosphate buffer (pH 8.5) before it was mixed with the 3HP solution at 1 M. The pH of the mixture was adjusted to 9.0 using 5 M NaOH, and the blend was allowed to react at room temperature for 20 min. This process was iteratively executed four times to achieve a final concentration of 3HP at 60 mM in the β‐LG mixture.

### 4.4 Plaque‐reduction assay to detect 3HP‐β‐LG‐mediated inhibition of VACV‐VTT and MPXV infection

The plaque‐reduction assay to detect 3HP‐β‐LG‐mediated inhibition of VACV‐VTT and MPXV infection was performed as previously detailed.^1^, ^30^ In brief, 2 × 10^4^ BHK‐21 cells (for VACV‐VTT) or 2 × 10^5^ Vero cells (for MPXV) were seeded into wells of a 96‐well (for VACV‐VTT) or 12‐well (for MPXV) plate a day before infection. 3HP‐β‐LG and β‐LG were diluted to the indicated concentrations and incubated with virus suspension (100 PFU/500 µL) at 37°C for 1 h before addition of the mixture to cells. Following 1‐h incubation at 37°C, the culture medium was changed. After incubation at 37°C for 48 h (for VACV‐VTT) or 96 h (for MPXV), the culture supernatant was discarded, and the cells were fixed with a solution containing 4% paraformaldehyde and 0.1% crystal violet for 2 h. The plaques were photographed and counted as previously described.^1^, ^30^ The percent inhibition of viral infection was calculated as (plaque number in a well containing virus only—plaque number in a well containing virus and inhibitor)/plaque number in a well containing virus only ×100%. Half maximal inhibitory concentration (IC_50_) was calculated with GraphPad Prism 10.0.

### 4.5 qPCR assay for detection of 3HP‐β‐LG‐mediated inhibition on VACV‐VTT and MPXV infection

The qPCR assay to evaluate VACV‐VTT and MPXV infectivity was adapted from the method previously described by Mills et al.^31^ Briefly, 2 × 10^4^ BHK‐21 cells (for VACV‐VTT) or 2 × 10^5^ Vero cells (for MPXV) were cultured with the mixture of 3HP‐β‐LG or β‐LG and virus as described above. After incubation at 37°C for 24 h (for VACV‐VTT) or 96 h (for MPXV), the wells of plates were blocked and stored at −80°C before detection of viral loads. Upon thawing the samples three times, total DNA extraction (TIANGEN; product #DP304) was conducted and then followed by quantitative PCR detection. Virus copy number was calculated based on a standard curve, and the percent inhibition of viral infection was calculated using the same formula as that described above, but replacing plaque number with viral copy number.

### 4.6 Cytotoxicity assay

The potential cytotoxic effect of 3HP‐β‐LG on BHK‐21 and Vero cells was assessed following the standard protocol detailed in the CCK‐8 manual.^16^ In brief, 3 × 10^4^ cells/well in a 96‐well plate were inoculated at 37°C overnight before addition of serially twofold diluted 3HP‐β‐LG. After incubation at 37°C for 24 h, the supernatants were replaced with fresh MEM culture medium containing 2% FBS. After further culture for 48–72 h, the supernatants were replaced with CCK‐8 solution (DOJINDO; product #CK04). Following a 2‐h incubation, the optical density at 450 nm (OD_450_) was measured using a microplate reader (SpectraMax^®^ i3x from Molecular Devices, USA). The percent cell viability was calculated as (OD_450_ for cells treated with 3HP‐β‐LG/OD_450_ for untreated control cells) × 100%.

### 4.7 Time‐of‐addition assay

BHK‐21 cells (3 × 10^4^/well) were inoculated in a 96‐well plate and cultured at 37°C throughout the night. 3HP‐β‐LG at 800 µg/mL was added to cells at 0.5 h before (−0.5 h), immediately (0 h), and 0.5, 1, 2, 3, 4, 5, and 6 h after addition of VACV‐VTT, respectively. Following a 2‐h interval post‐VACV‐VTT infection, the supernatant was removed, and MEM culture medium containing 2% FBS was added to the BHK‐21 cells. After culturing for 48 h at 37°C, the supernatants were removed, and the cells were fixed with a solution containing 4% paraformaldehyde and 0.1% crystal violet. The plaques were photographed and counted as described above.

### 4.8 Washout assay

To determine whether 3HP‐β‐LG acts on target cells, cells were treated with either 3HP‐β‐LG or β‐LG at 37°C for 1 h before they were washed with MEM to eliminate any unattached inhibitors before addition of VACV‐VTT. Inhibitory activity of 3HP‐β‐LG or β‐LG on VACV‐VTT was determined using the plaque‐reduction assay as described above.

### 4.9 Assays to study 3HP‐β‐LG's inhibitory mechanism of action against VACV‐VTT infection

To determine which stage of VACV‐VTT infection was targeted by 3HP‐β‐LG, a series of experiments were carried out as previously described.^16^ For the virus‐binding assay, a mixture of VACV‐VTT and 3HP‐β‐LG or β‐LG was added to BHK‐21 cells and incubated at 4°C for 1 h before cells were washed to remove unbound virus and inhibitor. For the postattachment assay, BHK‐21 cells were infected with VACV‐VTT at 4°C for 1 h. The cells were washed to remove the unbound virus, followed by addition of 3HP‐β‐LG. Following 1 h's incubation at 37°C, cells were washed to remove the unbound 3HP‐β‐LG. For the postentry assay, BHK‐21 cells were infected with VACV‐VTT at 37°C for 1 h before addition of 3HP‐β‐LG. After incubation at 37°C for one more hour, cells were washed to remove the unbound virus, followed by addition of 3HP‐β‐LG. After each of the above experiments, the inhibitory activity 3HP‐β‐LG on VACV‐VTT infection was detected as described above.

### 4.10 Determination of zeta potential

To determine the zeta potential of β‐LG protein modified with different ratios of anhydride 3HP, the modified β‐LG proteins were dialyzed against pure water for 60 h at 4°C. These anhydride‐modified proteins were then diluted to the same concentration (5 mg/mL) for measurement of zeta potential by Zetasizer (Nano ZS; Malvern Instruments Ltd., Malvern, WR14 1XZ, Worcestershire, UK) at room temperature (26°C). To ensure accurate readings, each sample was allowed an equilibrium time of 120 s before its zeta potential was recorded. For consistency and reliability, each sample's zeta potential was measured in triplicate, and the outcomes were expressed as means ± standard deviation (SD), as represented by error bars.

### 4.11 Expression and purification of MPXV surface protein A29L and others

The expression and purification of A29L were performed as previously described. Briefly, the MPXV A29L gene was synthesized and inserted into plasmid for expression. The engineered bacteria were cultivated in Lysogeny Broth (LB) supplemented with 50 µg/mL of kanamycin at a temperature of 37°C until they reached an optical density (measured at 600 nm, OD_600_) of 0.5. Induction was then initiated using 1 mM isopropyl‐beta‐d‐thiogalactoside at 16°C for 8 h. These bacteria were collected and resuspended in PBS containing 10 mM imidazole and 500 mM NaCl, and then lyzed via ultrasonic disruption conducted on ice. Thereafter, the lysate was separated by centrifugation and purified by Ni‐NTA (Smart‐Lifesciences, Changzhou, China). The same protocol was also used for expression and purification of other MPXV surface proteins, including A35R, B6R, E8L, H3L, and M1R.

### 4.12 Enzyme‐linked immunosorbent assay for detection of 3HP‐β‐LG binding to MPXV surface proteins

Binding of 3HP‐β‐LG to a MPXV surface proteins was detected by enzyme‐linked immunosorbent assay (ELISA) as previously discribed.^1^ In brief, wells of a 96‐well polystyrene plate were coated with a 15 µg/mL solution of 3HP‐β‐LG or β‐LG in PBS overnight at a 4°C,, and the plates were blocked with a 1% gelatin solution for 2 h at room temperature before addition of His‐tagged MPXV surface proteins A29L, A35R, B6R, E8L, H3L, or M1R at graded concentration. The plates were then incubated at 37°C for 1 h and washed with PBST (0.05% Tween‐20 in PBS) three times, followed by addition of the HRP‐conjugated anti‐His tag antibody (Thermo Fisher Scientific) at 1:3000 and substrate MTB sequentially for detection of OD_450_ using an enzyme‐labeled instrument (SpectraMax^®^ i3x, Molecular Devices, USA).

### 4.13 Detection of 3HP‐β‐LG binding to A29L with BLI

All BLI assays were conducted on the Octet RED96 instrument, maintaining a consistent shaking speed of 1000 rpm and a plate temperature set at 30°C throughout all operations. PBST served as the kinetic buffer for these experiments. To determine binding kinetics, the MPXV protein A29L conjugated with His‐Tag was immobilized onto anti‐His biosensors (Pall FortéBio) following activation. 3HP‐β‐LG or β‐LG was prepared in separate centrifuge tubes at designated dilution concentrations and then transferred into individual wells of a black polypropylene 96‐well microplate. The remaining wells were filled with PBST, while one row was reserved as a negative control containing only PBST. Each well had a total volume of 200 µL. The assay protocol consists of several stages. First, an initial baseline incubation was carried out in PBST for 60 s, followed by the immobilization of A29L at the concentration of 20 µg/mL onto the sensors for 100 s. Next, a binding step was performed whereby 3HP‐β‐LG or β‐LG interacted with the immobilized A29L for 300 to 600 s. Finally, a dissociation phase in PBST lasted 300 to 600 s, after which sensors were regenerated using a 10 mM glycine HCl solution. Using ForteBio Data Analysis software (version 9.0), the BLI data yielded interaction kinetics between the proteins.

### 4.14 Immunofluorescence detection of 3HP‐β‐LG‐mediated inhibition of A29L binding to Vero cells

The mixture of A29L (20 µg/mL) and 3HP‐β‐LG or β‐LG (800 µg/mL) was incubated with Vero cells for 1 h. After removal of the culture supernatant, the wells of plates were blocked with 100% carbinol for 15 min at 37°C, followed by washing cells with PBS three times. BSA (5%) and anti‐A29L hAb VACV‐301 (10 µg/mL)^4^ were added to cells. The plates were then incubated at 37°C overnight and washed with PBS three times before the addition of FITC‐conjugated anti‐human IgG (KPL; product #02‐10‐06) at 1:500 and incubation at 37°C for 1 h. After cells were washed three times with PBS, antifade mounting medium with DAPI was added to cells. About 8 min later, cells were photographed with a fluorescence microscope.

### 4.15 Combination study and synergy analysis

Tecovirimat and 3HP‐β‐LG combination study and synergy analysis were performed as previously described.^32^. Briefly, tecovirimat and 3HP‐β‐LG were mixed at a ratio of about 1:100 based on the potency of tecovirimat and 3HP‐β‐LG when they are tested individually. The mixture was serially twofold diluted and added to virus and cells for detection of anti‐VACV‐VTT activity of tecovirimat and 3HP‐β‐LG when tested alone and in combination using a plaque‐reduction assay as described above. Dose reduction (fold) was calculated by dividing the IC_50_ value of an inhibitor when it was tested alone by that of the same inhibitor tested in combination with another inhibitor. The CI was calculated by using CalcuSyn software.^33^ Strength of synergism is indicated by the CI values, that is, <0.1, 0.1∼0.29, 0.3∼0.69, 0.7∼0.84, and 0.85∼0.90, indicating very strong synergism, strong synergism, synergism, moderate synergism, and slight synergism, respectively.

### 4.16 Structural modeling analysis

The trimeric structure of the MPXV protein A29L protein was predicted using the default parameters of the Alphafold2 program.^34^ The structures of β‐LG (PDB entry 1BEB) were acquired from the Protein Data Bank. The predicted model of 3HP‐β‐LG was generated from β‐LG structure using COOT (https://www.ccp4.ac.uk/) as reported before.^16^ Modeling the interaction between 3HP‐β‐LG and A29L was performed with AutoDock (http://autodock.scripps.edu/). All structural figures were generated and analyzed using PyMOL (https://pymol.org/2/) and ChimeraX (http://www.rbvi.ucsf.edu/chimerax).

### 4.17 Statistical analysis

Statistically significant differences were analyzed by employing a paired *t*‐test using GraphPad Prism 10.0. Asterisks denote significance levels: *p < 0.05, **p < 0.01, ***p < 0.001, and ****p < 0.0001, while “ns” signifies no statistically significant difference.

## AUTHOR CONTRIBUTIONS

S. J., L. L., and W. T. conceived the idea and designed the research. Y. S., B. H., C. H., Y. Z., W. T., J. S., Y. L., A. X., and W. Q. performed the experiments. Y. S., B. H., C. H., and Y. Z. analyzed the data. S. J., L. L., Y. S., B. H., C. H., Y. Z, and W. T. wrote and revised the manuscript. All authors have read and approved the final manuscript.

## CONFLICT OF INTEREST STATEMENT

Shibo Jiang, Lu Lu, Yi'ou Sha, and Chen Hua are the inventors in the patent application regarding the 3HP‐β‐LG‐mediated anti‐MPXV activity. Shibo Jiang and Chen Hua are working in Shanxi Jinbo Bio‐Pharmaceutical Co., Ltd, but has no potential relevant financial or non‐financial interests to disclose. Other authors declare that they have no conflict of interest.

## ETHICS STATEMENT

Not applicable.

## Data Availability

All data used for the current study are available from the corresponding author upon reasonable request.
